# Antibiotic Resistance in the Alternative Lifestyles of *Campylobacter jejuni*

**DOI:** 10.3389/fcimb.2021.535757

**Published:** 2021-05-13

**Authors:** Daise Aparecida Rossi, Carolyne Ferreira Dumont, Ana Carolina de Souza Santos, Maria Eduarda de Lourdes Vaz, Renata Resende Prado, Guilherme Paz Monteiro, Camilla Beatriz da Silva Melo, Vassiliki Jaconi Stamoulis, Jandra Pacheco dos Santos, Roberta Torres de Melo

**Affiliations:** ^1^ Laboratory of Molecular Epidemiology, Faculty of Veterinary Medicine, Federal University of Uberlândia, Uberlândia, Brazil; ^2^ Laboratory of Cellular and Molecular Biology, Faculty of Veterinary Medicine, University of Uberaba, Uberaba, Brazil; ^3^ Multidisciplinary Laboratory, Department of Veterinary Medicine, Goiás University Center, Goiânia, Brazil

**Keywords:** campylobacteriosis, meropenem, SEM, tetracycline, biofilm

## Abstract

*Campylobacter jejuni* is the main pathogen identified in cases of foodborne gastroenteritis worldwide. Its importance in poultry production and public health is highlighted due to the growing antimicrobial resistance. Our study comparatively investigated the effect of five different classes of antimicrobials on the planktonic and biofilm forms of 35 strains of *C. jejuni* with high phylogenetic distinction in 30 of them. In the planktonic form, the existence of susceptible strains to colistin (7/35 – 20%) and resistance to meropenem (3/35 – 8.6%) represent a novelty in strains evaluated in Brazil. In biofilms formed with the addition of chicken juice, the number of resistant strains was significantly higher for colistin, erythromycin and meropenem (100%), but the susceptibility to tetracycline was shown as a control strategy for specific cases. High concentrations (1,060 ± 172.1mg/L) of antibiotics were necessary to control the biofilm structure in susceptible strains in the planktonic form, which is consistent with the high biomass produced in these strains. Stainless steel and polyurethane were the most (BFI=2.1) and least (BFI=1.6) favorable surfaces for the production of biomass treated with antimicrobials. It is concluded that the antimicrobial action was detected for all tested drugs in planktonic form. In sessile forms, the biomass production was intensified, except for tetracycline, which showed an antibiofilm effect.

## Introduction

*Campylobacter jejuni* is considered the main pathogen that causes human foodborne gastroenteritis worldwide, commonly found in the gastrointestinal tract of broilers. It is responsible for more than 500 million cases of diarrhea every year (CDC, 2020; EFSA, 2020) and in severe cases of infection by *C. jejuni*, individuals can develop post-infection complications such as Guillain Barré Syndrome (Goodfellow and Wilson, 2016).

Despite being considered a fastidious microorganism, *C. jejuni* has a high potential to produce biofilms, and thus survive and multiply in its hosts and in the environment (WHO, 2017a). This lifestyle provides greater adaptation to adverse conditions, including resistance to antimicrobials (Klein-Jobstl et al., 2016; Tang et al., 2017).

Biofilms are sets of microbial cells attached to biotic or abiotic surfaces involved in an extracellular matrix that significantly reduces susceptibility to antimicrobial agents when compared to planktonic cells. Sessile cell-related infections are, as a result, extremely difficult to treat. The low antimicrobials interaction with the biofilm matrix that prevents access to bacteria, reduction of multiplication rate and bacterial metabolism, and the intrinsic or plasmidial determinants of antibiotic resistance contribute to this profile and help ensure the survival of biofilm cells even under more aggressive antimicrobial treatment regimes (Hall and Mah, 2017). In addition, the presence of these structures in equipment and processing surfaces, such as stainless steel, polypropylene and polyurethane, become a persistent reservoir of contamination, compromising food safety and human health (Arnold and Silvers, 2000).

The indiscriminate use of antibiotics to fight infectious diseases has led to the emergence of many antibiotic-resistant bacteria, which have become a global problem in public health. Over the past years, several studies have reported these problem in *C. jejuni* strains (Melo et al., 2019; Elhadidy et al., 2020). For *C. jejuni*, the increase in the resistance profile over time is also associated with veterinary practices in the control of pathogens in birds. Antibiotics released in poultry production environments can interfere with the development of resistance profiles and affect the characteristics of bacterial biofilms or benefit the maintenance of the sessile life form, including *C. jejuni*, which produces highly stable and mature biofilms when in the chicken juice (CJ) presence (Melo et al., 2017).

Concerns are growing due to the current classification of *C. jejuni* by the WHO as a “high priority pathogen” due to the emergence of resistance to multiple drugs such as those belonging to the fluoroquinolone, macrolides, and other classes (Iovine, 2013; WHO, 2017b), which limits the treatment alternatives.

Given the impact of campylobacteriosis on public health, Brazil’s leading position as the world’s largest chicken meat exporter and the emergence of antimicrobial resistance in *Campylobacter* (Perio et al., 2013; EFSA-ECDC, 2015; WHO, 2017a; ABPA, 2020), it is necessary to constantly monitor the characteristics of this agent.

Our study investigated the differences in susceptibility to antibiotics, with different mechanisms of action, in *C. jejuni* isolated from chicken carcasses in planktonic and biofilm forms in the presence of CJ under different abiotic surfaces.

## Material and Methods

### Strains

Thirty-five strains of *C. jejuni* were used from the analysis from 442 carcasses of chilled or frozen chicken carcasses, ready for commercialization, isolated from September of 2015 to March of 2016, from the Brazilian poultry industry. The chickens were slaughtered in three different states (Minas Gerais, Goiás and Distrito Federal), in slaughterhouses authorized for export and under the supervision of the Federal Inspection Service.

The strains used were previously isolated and characterized by Melo (2017), following the ISO isolation protocols (International Standards Organization, 2006). Species identification was performed by multiplex PCR according to the protocol defined by Harmon et al. (1997), followed by maintenance at –80°C and reactivation, according to ISO (2006).

### Phylogenetic Analysis

The genetic similarity between the isolates was determined by the RAPD-PCR (random amplification of polymorphic DNA) technique in order to prove the phylogenetic distinction between the strains. We performed the analysis in three repetitions or until we obtained three identical results in order to guarantee the selection of more reliable data. The analysis was not possible in five of the 35 strains since the amplicons of DNA were not obtained for all strains using the selected primers, or it was not possible to obtain identical results for these strains.

Genomic DNA was extracted using the Genomic DNA Purification Wizard Kit (Promega, Madison, Wisconsin, USA), following the protocol established by the manufacturer. Purified DNA (10 ng) was used for the RAPD-PCR, which were performed with the HLWL85 (5’ACGTATCTGC3’) and 1290 (5’GTGGATGCGA3’) primers (Akopyanz et al., 1992; Mazurier et al., 1992). The RAPD-PCR technique was performed according to Akopyanz et al. (1992) until three repetitions were obtained with identical results for the same strain. The reaction was prepared in a total volume of 20 μL, composed of 10 ng/µl of bacterial DNA, 10 mM of Tris-HCL; 50 mM of KCl; 2.0 mM of MgCl_2_ and 1U of Taq DNA polymerase (Invitrogen^®^, Waltham, Massachusetts, USA); 200 µM of each triphosphate deoxynucleotide (DNTP) (Invitrogen^®^, Waltham, Massachusetts, USA) and 30 picomoles of the primer (Invitrogen^®^, Waltham, Massachusetts, USA).

The amplification occurred under the following conditions: 1 initial denaturation cycle at 92°C for 2 minutes; 35 cycles of three stages: denaturation at 92 °C for 15 seconds, annealing at 36°C for 1 minute, extension at 72°C for 1 minute; and one final extension cycle at 72°C for 5 minutes.

The amplified products were submitted to electrophoresis on 1.5% agarose gel (Affymetrix^®^, Santa Clara, California, USA), using TBE 0.5X running buffer (Invitrogen^®^, Waltham, Massachusetts, USA) and the 100 bp marker (Invitrogen^®^, Waltham, Massachusetts, USA) as the molecular weight standard. The gel was stained with Syber Safe (Invitrogen^®^, Waltham, Massachusetts, USA), visualized and captured in a transilluminator (Loccus Biotecnologia, Cotia, São Paulo, Brazil).

The dendrogram was built using the GelCompar II software (Comparative Analysis of Electrophoresis Patterns), version 1.50. The comparison of the band patterns was performed by the UPGMA method (unweighted pair group method with arithmetic mean), using the Dice similarity coefficient. Strains considered similar were only those that showed 95% of homology due to the discriminatory power of the technique.

### Planktonic Cells

After the reactivation of strains on CCDA agar (*Campylobacter* Blood-Free Selective Agar Base) (Oxoid, Basingstock, Hampshire, UK), isolated colonies were introduced in 2 mL of 85% NaCl (Synth^®^, Diadema, São Paulo, Brazil) and the standardized concentration was 0.5 on the McFarland scale, corresponding at 5.5 log CFU/mL (EUCAST, 2020) to determine the antibiotic susceptibility testing (*Antibiotic Susceptibility Testing*).

### Biofilm Formation (Traditional Method)

For the biofilm formation, initially, the cultures present in the CCDA plates were transferred to 20 mL of Mueller Hinton broth (MH) (Difco, Sparks, Maryland, USA) supplemented with 5% of CJ and incubated at 37°C for 48 hours under microaerophilia. To simulate the nutritional conditions of abiotic surfaces present during processing in poultry industry and to guarantee qualitative and quantitative stability of the biofilm (Melo et al., 2017), we used the model system with 5% CJ, equivalent to 100% concentration according to Brown et al. (2014), based on supplementation of the culture medium with thawed poultry exudates sterilized by filtration (Birk et al., 2006).

After growth, the bacterial suspension was standardized to an OD_600_ = 0.22 to 0.28 and centrifuged at 5000 rpm for 10 min at 4°C. After discarding the supernatant, the cells were washed and centrifuged twice in 0.9% sterile NaCl solution. The supernatant was discarded, and the pellet was resuspended in 0.9% NaCl solution and diluted in 10 mL of supplemented MH broth in order to obtain a final count of 10^4^ CFU/mL.

The technique of biofilm formation was performed according to Sulaeman et al. (2009), with modifications. Briefly, 200 µL of the bacterial suspension in MH with 5% of CJ containing 10^4^ cells was added in 96-well plates and incubated for 48 hours at 37°C under microaerophilic conditions. Afterwards, the non-adherent bacteria were washed twice with 0.9% sterile NaCl solution and the biofilm formed, corresponding to an average of 6.14 ± 0.52 log CFU/mL, was maintained for treatment with antibiotics, as described in *Antibiotic Susceptibility Testing*.

### Antibiotic Susceptibility Testing

After preparing the suspension of free bacteria (*Planktonic Cells*) and the production of biofilms in the microplates [*Biofilm Formation (Traditional Method*)], the antimicrobial susceptibility of 35 strains in planktonic and biofilm forms was determined against ciprofloxacin (fluoroquinolone that acts on bacterial DNA replication), erythromycin (macrolide that inhibits protein synthesis and translation), tetracycline (tetracycline inhibiting protein synthesis), meropenem (carbapenem that inhibits cell wall synthesis) and colistin (polymyxin that acts in the destructuring bacterial cell membrane) (Abushaheen et al., 2020), the latter being little studied due to considering resistance as intrinsic to the pathogen. The method used was the broth microdilution in microplates (Kasvi, São José dos Pinhais, Brazil), as described in EUCAST (2020), following the specifications and cutoff points for *Campylobacter* and *Enterobacteriaceae*, when applicable.

The criterion for choosing these antimicrobials was based on the use of this drug in veterinary and human medicine and because of their different mechanisms of action. The tested concentrations were: 0.125, 0.25, 0.5, 1, 2, 4, 8, 16, 32, 64, 128, and 256 µg. mL^-1^.

For the planktonic form, twenty microliters of the bacterial suspension were transferred into 180 μL cation-adjusted (20–25 mg/L Ca^2+^, 10–12.5 mg/L Mg^2 +^) MH with antimicrobial and 5% lysed sheep blood (LaborClin, Pinhais, Paraná, BR). For biofilms already present in microplates, we only transferred 200 μL cation-adjusted (20–25 mg/L Ca^2+^, 10–12.5 mg/L Mg^2+^) MH with antimicrobial and 5% lysed sheep blood (LaborClin, Pinhais, Paraná, BR). The multiwell plate was sealed and incubated at 41 ± 1°C for 40–48 h in microaerobic conditions. In addition, a 10 μL aliquot of each diluted inoculum was plated in CCDA agar (Oxoid, Basingstock, Hampshire, UK) to check the bacterial growth (viability) of the respective dilution well.

For all tests, negative controls composed of the medium without the addition of bacteria were used. For the tests performed for colistin, *E. coli* ATCC 25922 and *E. coli* NCTC 13846 strains were used as positive controls, the latter being positive for the *mcr1* gene, as recommended in the EUCAST manual (2020), and for the other determinations, the strains *C. jejuni* IAL 2383 and NCTC 11351 were used as positive controls.

### Biomass Analysis

**Figure 1:**
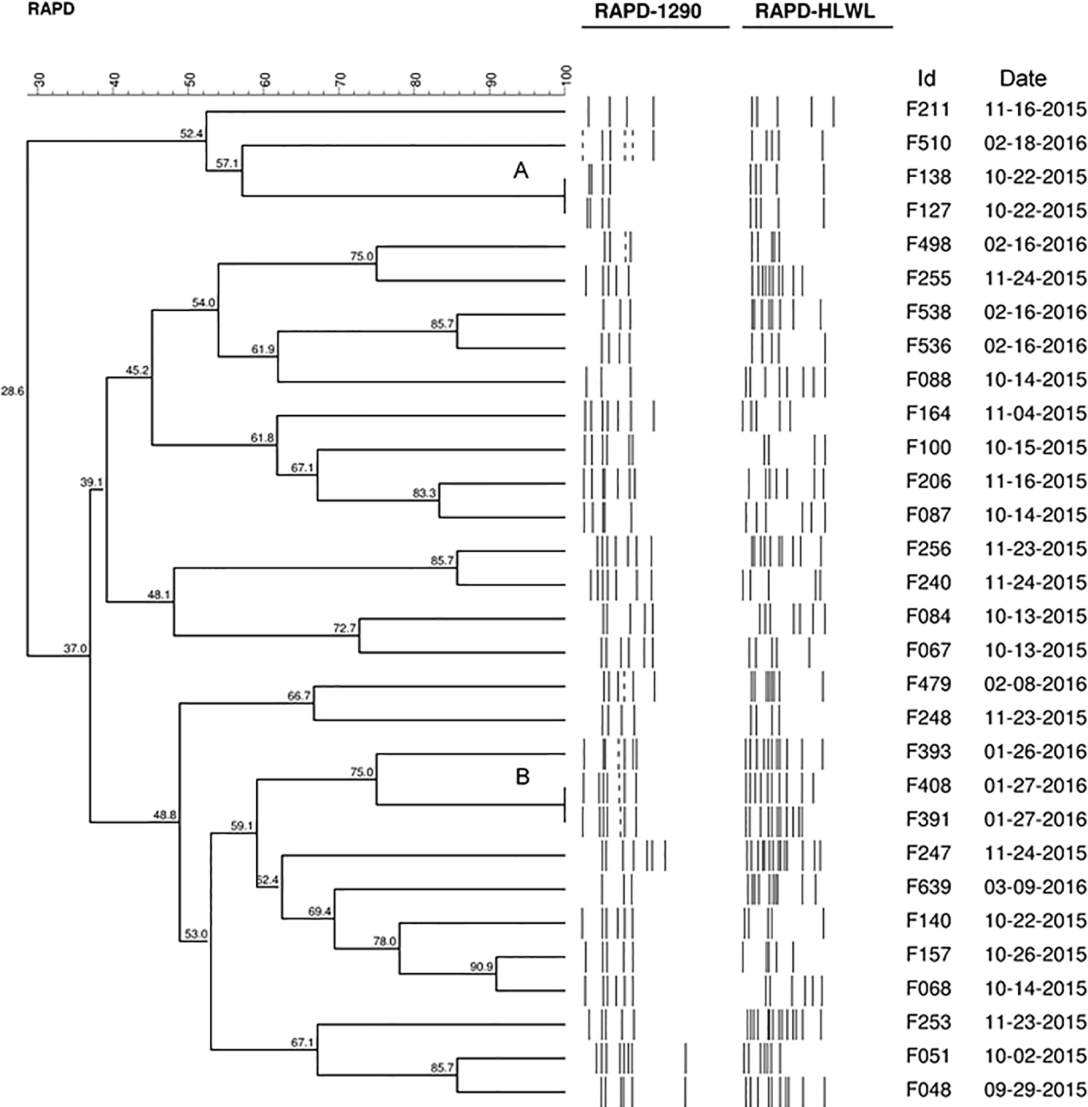
Dendrogram generated by computerized analysis (Gel Compare II) of DNA profiles of 30 strains of *C. jejuni*, based on RAPD-PCR. The analysis was performed using the Dice/UPGMA method (0.5% tolerance parameter, 0.5% optimization, homology ≥95%).

Qualitative analysis of biofilm biomass with (tests) and without (control) antimicrobial treatment at a concentration of 32 µg.mL^-1^ was performed on two strains of *C. jejuni*, previously characterized in terms of the resistance profile in this study (F048 and F639). The strains were selected considering their differences linked from origin industry, isolation years (2015 and 2016, respectively), phylogenetic distance (53% of similarity – **Figure 1**) and previous antimicrobial susceptibility testing results in planktonic form (the first susceptible to tetracycline and erythromycin and the second pan-resistant - **Table 1**) and sessile form (F048 susceptible to tetracycline and F639 pan-resistant - **Table 1**). This method was adopted to verify the variation of biomass (sessile bacteria + extracellular matrix) in biofilms produced by *C. jejuni* without and with antibiotic treatment. The concentration of antibiotic used was defined based on preliminary results that demonstrated MIC ≥32µg.mL^-1^ for all tested antibiotics, except tetracycline, being one strain susceptible and the other resistant to tetracycline in biofilm form (**Table 2**).

**Table 1:** MIC of biofilm and planktonic forms of two strains of *C. jejuni* used in biomass and imaging tests.

Strains	MIC in planktonic form		MIC in biofilm form	
	Antimicrobial (mg/L) S/R		Antimicrobial (mg/L) S/R	
F048	CIP (4) R	COL (16) R	CIP (>256) R	COL (256) R
	TET (<0.125) S	ERY (2) S	TET (2) S	ERY (256) R
	MER (1) S		MER (>256) R	
F639	CIP (64) R	COL (256) R	CIP (256) R	COL (>256) R
	TET (128) R	ERY (64) R	TET (>256) R	ERY (>256) R
	MER (64) R		MER (>256) R	

The biofilms produced in multiwell plates, according to *Biofilm Formation (Traditional Method)*, were treated with five classes of antimicrobials at a concentration of 32µg.mL^-1^ prepared in cation-adjusted (20–25 mg/L Ca^2+^, 10–12.5 mg/L Mg^2+^) MH with 5% lysed sheep blood (LaborClin, Pinhais, Paraná, BR). For control, we use only cation-adjusted (20–25 mg/L Ca^2+^, 10–12.5 mg/L Mg^2+^) MH with 5% lysed sheep blood (LaborClin, Pinhais, Paraná, BR) without antimicrobial. The multiwell plates were incubated as described in EUCAST (2020).

After obtaining and treating of the biofilms, the media was removed, the wells were washed twice with 0.9% NaCl solution and dried for 30 minutes at 55°C. The total biomass was measured by fixing with 0.1% Violet Crystal (LaborClin, Pinhais, Paraná, BR) for 5 minutes, followed by elution with methanol solution (Synth^®^). The eluted dye was evaluated by reading at the OD_595_. The tests were performed with eight replicates for each strain in three replications.

To determine the Biofilm Formation Index, the following formula was used: BFI = (BA – PC)/BS, where BFI represents the result referring to the Biofilm Formation Index, BA the optical density obtained in the mixture of adhered bacteria, PC the value of the absorbance in microorganism-free control wells, BS the optical density of cultures in suspension (Naves et al., 2008). For the BFI classification were considered, ≥1.10 = strong, 0.70–1.10 = medium, 0.35–0.69 = weak, and <0.35 = non-existent.

### Biomass Analysis in Different Materials

For the biofilm production by the two selected strains (F048 and F639), the tests were performed in triplicate in three independent periods. The tests were performed on 1cm^2^ slides of the following materials: polyurethane (PU) (Habasit Cleandrive TM, Reinach, CH), polypropylene (PP) (Leadmec, Belo Horizonte, Minas Gerais, BR), and stainless steel (AISI 304).

The initial inoculum for biofilm production was 6.66 ± 0.28 log CFU/mL, corresponding to DO600nm of 0.22 to 0.28, obtained in bacterial suspension in MH broth with 5% of CJ. The inoculum was then used to produce biofilms on slides 48 hours at 37°C under microaerophilic conditions. Three slides of each material (for each strain and for each antimicrobial) were aseptically transferred to a 24-well polystyrene plate (Kasvi, São José dos Pinhais, Paraná, BR) in each repetition. After biofilm formation, we determined the effect of the five antimicrobials on the biofilms produced. The slides were aseptically transferred to 24-wells polystyrene plates (Kasvi, São José dos Pinhais, Paraná, BR) and treated with five classes of antimicrobials at a concentration of 32µg.mL-1 prepared in cation-adjusted (20–25mg/L Ca^2+^, 10–12.5 mg/L Mg^2+^) MH with 5% lysed sheep blood (LaborClin, Pinhais, Paraná, BR). For control, we use only cation-adjusted (20–25 mg/L Ca^2+^, 10–12.5 mg/L Mg^2+^) MH with 5% lysed sheep blood (LaborClin, Pinhais, Paraná, BR) without antimicrobial. The 24-wells polystyrene plates were incubated as described in EUCAST (2020).

After incubation, these slides were washed twice with 0.9% NaCl solution, transferred to a new 24-well polystyrene plate, and dried for 30 minutes at 55°C. The total biomass was measured by fixing with 0.1% Violet Crystal (LaborClin, Pinhais, Paraná, BR) for 5 minutes, followed by elution with methanol solution (Synth^®^). The dissolved dye was removed from each slide and placed in a new 96-wells microtiter plate for OD_595_ reading on a spectrophotometer (DNM-9602 microplate reader Perlong). The biomass classification of biofilms was performed according to the protocol described by (Naves et al., 2008), described in *Biomass Analysis*.

### Scanning Microscopy

The visualization of the biomass formed with (tests) and without (control) antimicrobial treatments at a concentration of 32 µg.mL^-1^ was performed in a scanning electron microscope, with two strains of *C. jejuni*, one susceptible and the other resistant to tetracycline in a biofilm form (F048 and F639). The preparation of the material for analysis in the SEM was done according to Brown et al. (2014), with modifications. Biofilms were formed in glass beads with a diameter of 5 mm, respecting the growth conditions described above. After biomass formation, the samples were fixed with 2.5% glutaraldehyde and 2.5% paraformaldehyde in 0.1M PBS buffer (pH 7.4) overnight at 4°C. The fixative was removed, and the samples were washed three times with PBS buffer. The beads were post-fixed with 1% osmium tetroxide for two hours and washed three times with PBS buffer. The beads were dehydrated in a series of ethanol solutions (30, 40, 50, 60, 70, 80, and 90% and then three times at 100%) for 15 minutes for each step.

The samples were dried on CDP (critical drying point) (CDP 030, Baltec, DE) using liquid carbon dioxide as the transition fluid, then coated with a 20nm thick gold layer (SCD 050, Baltec, DE) and visualized in SEM VP Zeiss Supra 55 FEG SEM operating at 20 kV.

### Statistical Analysis

The test results were submitted to descriptive statistics, normality analysis, followed by application of the T student test/Mann-Whitney test (for comparisons of results referring to concentrations necessary for the control biofilms), Fisher’s exact test (used in results comparisons obtained for planktonic and biofilms forms), ANOVA/Kruskal-Wallis (in the interpretation of data obtained in biomass tests). A significance level of 5% was adopted, using the Graph Pad Prism 8.0.1 Program for the calculations.

## Results

### Genetic Proximity

Using 95% of similarity values, RAPD-PCR revealed significant differences in the 30 strains of *C. jejuni* population evaluated and, therefore, they were considered different strains. The exception was for two clusters with 100% of similarity (A and B) detected in the dendrogram produced by the analysis of the 30 strains (**Figure 1**). Cluster A included strains F127 and F138 resistant to ciprofloxacin, colistin, and tetracycline, and cluster B included strains resistant to ciprofloxacin and tetracycline (F391 and F408). In addition, in biofilms, all these strains showed resistance to all the studied drugs.

### Resistance of Planktonic *C. jejuni*

Our study found resistance percentages ≥80% for ciprofloxacin (31/35, 88.6%), tetracycline (30/35, 85.7%), and colistin (28/35, 80.0%) in planktonic form. The highest susceptibility was attributed to meropenem (32/35, 91.4%) and erythromycin (23/35, 65.7%), therefore, considered the most efficient drugs (**Table 2**).

Low concentrations were sufficient for MIC_50_ of meropenem (0.25 mg/L) and erythromycin (0.5 mg/L), whereas for tetracycline, ciprofloxacin, and colistin the values were 32, 128, and 256 mg/mL, respectively. MIC_90_ was 8 and 256 mg/L for meropenem and erythromycin, respectively; for the other antibiotics, the concentration was higher than 256 mg/L (**Table 2**).

**Table 2:** MIC, MIC_50_, MIC_90,_ and resistance rate distributions for investigated of 35 C*. jejuni* strains in biofilm and planktonic forms.

Antibiotics (mg/L)	CIPPL	CIPBF	COLPL	COLBF	TETPL	TETBF	ERYPL	ERYBF	MERPL	MERBF
<0.125	01	–	–	–	04	–	08	–	10	–
0.125	01	–	–	–	–	–	–	–	05	–
0.25	02	–	02	–	01	–	04	–	05	–
0.5	–	–	03	–	–	–	06	–	01	–
1	01	-	02	–	–	02	01	–	02	–
2	01	-	–	–	–	01	02	–	03	–
4	03	-	01	-	03	01	02	–	03	–
8	01	-	-	-	01	02	–	–	03	–
16	02	-	01	-	06	02	02	-	-	-
32	03	-	04	01	06	02	01	01	02	-
64	02	-	-	02	07	03	02	-	01	-
128	02	03	03	03	01	08	03	-	-	02
256	06	11	02	04	01	03	04	07	-	06
>256	10	21	17	25	05	11	-	27	-	27
Total Resistant n (%)	31 (88.6)	35 (100)	28* (80)	35* (100)	30 (85.7)	32 (91.4)	12** (34.3)	35** (100)	03** (8.6)	35** (100)
Total Resistant ≥32 – n (%)	23* (65.7)	35* (100)	26* (74.3)	35* (100)	20 (57.1)	27 (77.1)	10** (28.6)	35** (100)	03** (8.6)	35** (100)
MIC_50_	128	>256	256	>256	32	128	0.5	>256	0.25	>256
MIC_90_	>256	>256	>256	>256	>256	>256	256	>256	8	>256

PL, planktonic form; BF, biofilm form; CIP, ciprofloxacin; COL, colistin; TET, tetracycline; ERY, erytromycin; MER, meropenem; ___, breakpoint (EUCAST, 2020); n = resistant strains number; % = Resistance rate; *p < 0.05, **p < 0.0001 using Fisher’s exact test.

We identified 11 resistance profiles, 14.3% (5/35) of whom presented co-resistance. Multiple antimicrobial resistance (MDR) (resistance to three or more classes of antimicrobials) was presented in 71.4% (25/35) and included six distinct profiles, two (P6 and P7) grouping 12 strains resistant to three classes, two (P8 and P9) with 12 other strains resistant to four classes and one profile (P11) with a strain resistant to all tested classes. The most frequent profile was P6, which included 11 of the 35 strains (31.4%) and indicated joint resistance to ciprofloxacin, colistin, and tetracycline, followed by P8 with 28.6% (10/35) of strains resistant to the same antibiotics and also erythromycin. If we consider the intrinsic resistance to colistin, the total of multiresistant strains was 13/35 (37.1%) (**Table 3**).

### Resistance of *C. jejuni* Biofims

In general, our results showed that the tested drugs had no effect on the biofilm structure of *C. jejuni*. Only for tetracycline was it possible to detect some exceptions.

The sessile cells in biofilms of the 35 strains of *C. jejuni* were resistant to ciprofloxacin, erythromycin, colistin, and meropenem, requiring concentrations equal to or greater than 32 mg/L to inhibit them. The exception is tetracycline, in which three strains (8.6%) were susceptible according to the breakpoint defined by EUCAST (2020). All strains (100%) in biofilms were resistant to erythromycin, meropenem, and colistin, showing that there was a significant increase compared to the number of resistant strains in planktonic form (12/35; 3/35; and 28/35, respectively). When the analysis was restricted to concentrations ≥32 mg/L of the antibiotic, only for tetracycline we did not observe a difference in both forms of life (20 plactonic strains and 27 sessile strains) (**Table 1**). In addition, for tetracycline alone, we detected an average of 19-fold reduction in MIC value in four strains (11.4%) in biofilms.

The MIC_50_ and MIC_90_ of tetracycline were 128 and >256 mg/L, respectively. For the other antimicrobials, this value was >256 mg/L for both MIC_50_ and MIC_90_ (**Table 2**).

The need for high concentrations of antimicrobials to control sessile cells of *C. jejuni* in biofilms showed the highest number of multidrug-resistant strains, found in all strains in profiles P10 (CIP-COL-ERY-MER) detected in 3/35 (8.6%) strains and P11 (all classes) in 32/35 (91.4%) strains (**Table 3**).

We consider concentrations >256 and <0.125 mg/L equal to the values immediately above and below, 512 and 0.0625 mg/L, respectively, to allow the quantitative analysis of all strains. The variation in the concentration of antibiotic needed to inhibit the sessile cells in biofilm was strain-dependent for all tested antibiotics, and this characteristic was maintained when separately evaluating susceptible and resistant strains (p <0.05). Susceptible strains needed an average concentration of 2,595 ± 353.4 times higher of antibiotics to inhibit sessile cells compared to planktonic bacteria, with no significant difference between the drugs tested (p = 0.0956). This value was significantly lower for resistant bacteria and equivalent to 12.6 ± 3.3 and statistically equal for all antibiotics (**Table 4**).

**Table 3:** Resistance profiles in 35 Campylobacter jejuni isolated from poultry meat.

Antimicrobial agents	Planktonic N (%)	Biofilm N (%)
(P1) CIP	1	–
(P2) COL	2	–
(P3) TET	2	–
**Total mono-resistant isolates**	**5 (14.3)**	**0**
(P4) CIP-TET	4	–
(P5) CIP-COL	1	–
**Total co-resistant isolates**	**5 (14.3)**	**0**
(P6) CIP-COL-TET	11	–
(P7) CIP-COL-ERY	1	–
(P8) CIP-COL-TET-ERY	10	–
(P9) CIP-COL-TET-MER	2	–
(P10) CIP-COL-ERY-MER	–	3
(P11) CIP-COL-TET-ERY-MER	1	32
**Total MDR isolates**	**25 (71.4)^a^**	**35 (100.0)^b^**

CIP, ciprofloxacin; COL, colistin; TET, tetracycline; ERY, erytromycin; MER, meropenem; N (%), total number and percent of C. jejuni isolates; P, profile; Superscript letter ^(a or b),^ distinct letters in the same row indicate that numbers are statistically different (Fisher’s exact test). The final value referring to the previous lines and the option in bold highlights the information.

**Table 4:** The average increase in antimicrobial concentrations necessary for the inhibition of biofilms in 35 strains of *C. jejuni* compared to MIC in planktonic form.

Antimicrobial	CIP	COL	TET	ERY	MER	General average
**All strains**	314.4 ± 234.3^A^	96.5 ± 59.1^A^	71.1 ± 58.4^A^	1,826 ± 450.3^B^	2,994 ± 542.1^B^	1,060 ± 172.1
**Susceptible**	2,560 ± 1,881^Aa^	466.3 ± 265.2^Aa^	448 ± 400.5^Aa^	2,772 ± 598.5^Aa^	3,274 ± 568.5^Aa^	2,595 ± 353.4^Aa^
**Resistant**	24.7 ± 9.8^Ab^	4 ± 1.4^Ab^	8.6 ± 4.7^Ab^	11.7 ± 3.7^Ab^	13.3 ± 2.7^Ab^	12.6 ± 3.3^Ab^

CIP, ciprofloxacin; COL, colistin; TET, tetracycline; ERY, erythromycin; MER, meropenem; Different uppercase letters on the lines indicate a significant difference, different lowercase letters on the columns indicate a significant difference (Kruskal–Wallis test).

In the evaluation of all strains, we observed that there was no difference in the average increase in the concentrations of ciprofloxacin, colistin, and tetracycline necessary to inhibit sessile cells (**Table 4**), probably because we had a greater number of resistant strains for these antibiotics (**Table 2**). Similarly, for erythromycin and meropenem, as we had a greater number of susceptible strains. In an analysis that includes only the precise values obtained in the MIC, with the concentration range evaluated (0.125, 0.25, 0.5, 1, 2, 4, 8, 16, 32, 64, 128, and 256 mg/L), we detected that susceptible strains needed an average concentration of 376.3 ± 240.2 times higher of antibiotics, regardless of class (p = 0.4414), to control the sessile cells in biofilm form. Resistant strains, on the other hand, needed an average concentration 24.74 ± 26.53 (p = 0.4475) times higher, a value significantly lower than that observed for susceptible bacteria (p = 0.0285, *t-Test*).

### Analysis of Biomass of *C. jejuni* Biofilms

Biomass tests, as well as imaging tests, were performed only with strains F048 and F639. The characterization of the antimicrobial resistance profile for these two strains is described in **Table 1**.

**Figure 2:**
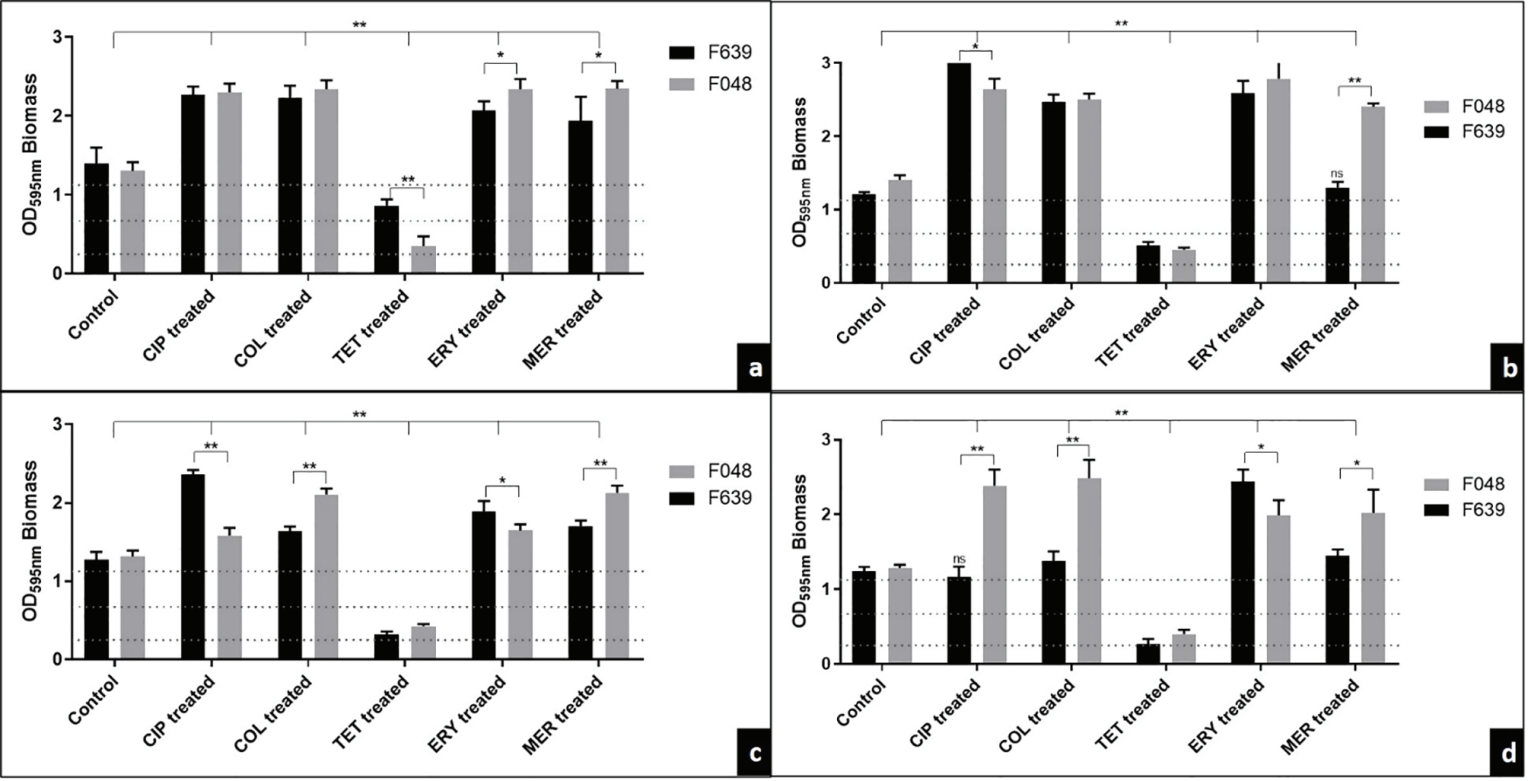
The effect of antibiotic treatment on biofilms supplemented with CJ from two strains of *C. jejuni* by the traditional method in 96-well microplates **(A)**, on stainless steel **(B)**, polypropylene **(C)**, and polyurethane **(D)** surfaces. Results represent means with standard deviation (error bars) of three independent experiments with eight replicates. CIP, ciprofloxacin; COL, colistin; TET, tetracycline; ERY, erythromycin; MER, meropenem; Treated, treatment with 32 mg/L of the antibiotic;…. BFI classification limits. ns, not significant in relation to the control; *p <0.05; **p <0.001 using one way ANOVA.

Both strains were classified as strong producers of biofilms in the BFI in the control group in the microplate test and for the three different surfaces tested (1.303 ± 0.025) (**Figure 2**). After treatment with ciprofloxacin, colistin, erythromycin, and meropenem, we observed a significant mean increase of 0.877, 1.162, 0.585 and 0.654 in the biomass intensity in the traditional test and on stainless steel, polypropylene and polyurethane surfaces, respectively. In contrast, treatment with tetracycline reduced the biofilm classification to low intensity in strain F048 and medium intensity to F639, in the traditional method (mean = -0.751), and for low intensity in both strains on the three surfaces (mean = -0.893).

For the traditional method, we observed that the sensitivity of F048 to erythromycin and meropenem detected in the planktonic form promoted a greater stimulus in the production of biomass when compared with F639 (pan-resistant strain) (mean = +0.27 and +0.41, respectively) (**Figure 2A**). This same profile was maintained in the biofilms produced on the different surfaces exposed to meropenem (mean = +1.10, +0.42, and +0.58, respectively to stainless steel, polypropylene and polyurethane) (**Figures 2B–D**).

In a different way, tetracycline promoted a significant reduction in the F048 biomass (susceptible in planktonic and biofilm forms) compared to F638, for traditional analysis (mean = -0.51). The same behavior observed in biofilms treated with ciprofloxacin and colistin was expected since both strains were resistant to these drugs (**Figure 2A**). On the different surfaces tested, we detected significant fluctuations in the biomass produced in contact with the antimicrobials, consistent with a strain-dependent character. The exception is tetracycline, whose BFI profile has not changed according to the type of surface (0.36 to 0.51 = weak BFI) (**Figures 2B–D**).

**Figure 3:**
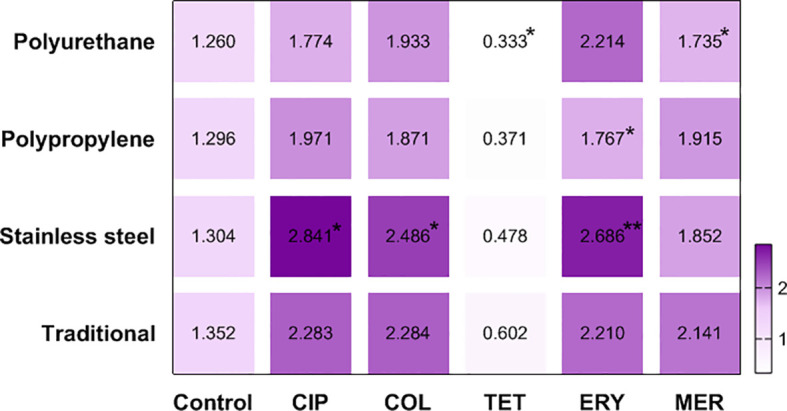
Heat map defined by the average BFI of two strains of *C. jejuni* in different materials after antimicrobial treatment. CIP, ciprofloxacin; COL, colistin; TET, tetracycline; ERY, erytromycin; MER, meropenem; *p <0.05; **p <0.001 using Kruskal-Wallis test (GraphPad Prism 8.0.1 software).

The heat map made it evident that stainless steel intensified the production of biomass treated with ciprofloxacin (BFI = 2.841), colistin (BFI = 2.486) and erythromycin (BFI = 2.686). Polyurethane was the least favorable surface for biofilms treated with tetracycline (BFI = 0.333) and meropenem (BFI = 1.735), as well as polypropylene in biofilms in contact with erythromycin (BFI = 1.767) (**Figure 3**).

### Image Analysis of *C. jejuni* Biofilms

**Figure 4:**
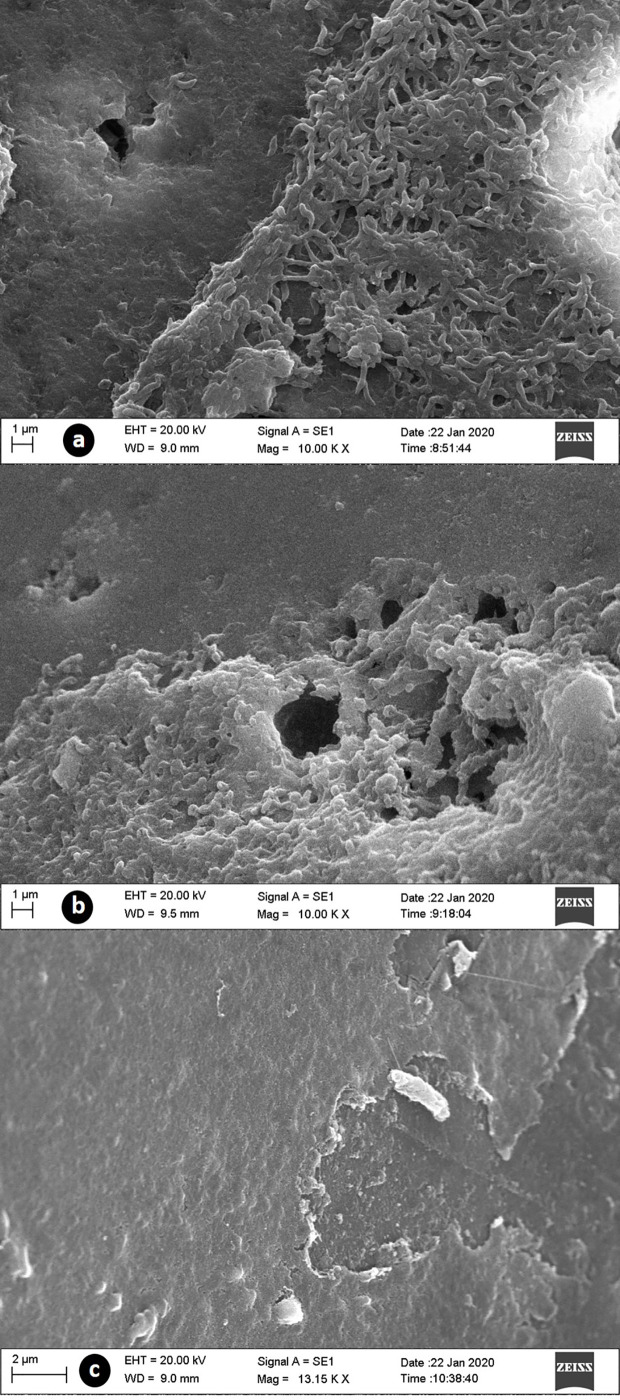
SEM images of biofilms supplemented with CJ from *C. jejuni* treated with antibiotics at 32 mg/L. **(A)** control group with normal biofilm structure; **(B)** treated with meropenem, demonstred the presence of a thick layer of extracellular matrix; **(C)** treated with tetracycline, with razing of the matrix structure and bacterial exposure.

In the SEM of strains F048 and F639, we observed the formation of mature biofilms with an evident three-dimensional structure of the matrix, however, three distinct characteristics were detected. The first one concerned an expanded architecture, compact matrix, large pores, and bacteria exposed on the surface (control). In the second morphology, we found a layered arrangement showing an increase in the three-dimensional structure, compact matrix, pores of smaller size, and absence of exposed bacteria in the superficial portion (treatments with ciprofloxacin, colistin, erythromycin, and meropenem). The last case concerned a biofilm in the death or disintegration phase, with matrix destruction, absence of pores, bacterial exposure, and evidence of cell death (treatment with tetracycline) (**Figure 4**).

## Discussion

### Genetic Diversity

The genetic diversity of 30 from 35 strains of *C. jejuni* was assessed using RAPD-PCR. Although RAPD-PCR is not the gold standard for genotyping due to low reproducibility, it is a cheap, easy, and discriminatory typing method to investigate the genome variability of a large isolates number, particularly for *C. jejuni*, which is a microaerobic and fastidious organism (Siddiqui et al., 2015).

Our study found a high molecular distance between the strains, a result already expected for *C. jejuni* due to its genetic plasticity. Most strains of *C. jejuni* are naturally competent for the capture of external DNA, so that recombination through transformation becomes a constant event, being the main driver of genetic diversity in this species. This ability resulted in a highly variable clonal population structure so that the boundaries between different groups of related genotypes were difficult to determine (Meric et al., 2014).

In poultry production, bacterial populations are constantly under strong pressure due to temperature variations, the use of antimicrobials, and health control actions. In addition to the high recombinant potential, *C. jejuni* is a bacterium that is phase-variable (PV), which allows the generation of specific genotypes depending on the niche. This happens due to the instability of the polyG regions, whose mutations allow the realization of rearrangements in the genome and the mobility of fragments (Bayliss et al., 2012), contributing to the observed genetic diversity.

### Antimicrobial Resistance Profile in the Planktonic Form

The alarming concern with antimicrobial resistance has mobilized the various public health entities globally to better understand the problem and to develop strategies of control, especially for *C. jejuni*. The available antibiotics are becoming less effective, and the high and increasing rates of resistance not only represent an obstacle to the prevention and treatment of the disease, but also increase the cost of health care (Akinkunmi et al., 2014; Elhadidy et al., 2020).

Our findings show that the situation is even more dramatic for ciprofloxacin and tetracycline, with more than 85% of resistant strains. Ciprofloxacin is a fluoroquinolone of choice for the treatment of campylobacteriosis, however, it is often prescribed as part of empirical treatments for undifferentiated diarrhea in humans. In addition, the use of fluoroquinolones in the late 1990s was extensively expanded in animal husbandry (Iovine, 2013; Kaakoush et al., 2015). Due to the problems associated with antimicrobial resistance, a ban on the use of this class of antimicrobial in Brazil has been enacted since 2009 as a growth promoter or as a preventive medication, but it is still allowed for therapeutic purposes. The use of this drug can promote the selection of resistant bacterial strains in the digestive tract of chickens, which justifies the high percentage of resistance identified and the concordance with results reported in several countries (Di Giannatale et al., 2019; Ilktac et al., 2019; Li et al., 2019).

The class of tetracyclines has been suggested as an alternative drug in the treatment of campylobacteriosis. However, several studies have demonstrated its low efficiency due to the dissemination and easy acquisition of genes for resistance to this antibiotic in the genus *Campylobacter*. The gene that encodes the *tetO* ribosomal protection protein of plasmid origin is considered the main responsible for the resistance to tetracyclines, displacing tetracycline from its primary binding site on the ribosome (Elhadidy et al., 2020).

For colistin, the existence of susceptible strains (7/35, 20%) represents a relevant and discrepant finding in the literature since it is already defined that *Campylobacter* exhibits intrinsic resistance to polymyxin/colistin, probably due to the absence of appropriate targets and/or low affinity of binding to targets (Iovine, 2013). Despite this finding, some studies have also identified the existence of *Campylobacter* susceptible to the class of polymyxins (Ghimire et al., 2014; Komba et al., 2015; Khoshbakht et al., 2016). Sorlózano-Puerto et al. (2018) found values low enough (range: 0.38–8 mg/L) for colistin sulfate to be considered useful in the treatment of severe diarrhea caused by *Campylobacter* spp. in an evaluation of 30 strains in Spain. Oral treatment with colistin is indicated in cases of enterocolitis by Gram-negative bacteria, such as pathogenic *E. coli* because colistin sulfate is poorly absorbed by the gastrointestinal tract and can reach high concentrations in the intestinal lumen (Li et al., 2005). However, there are restrictions on the use of this medicine in human medicine due to its classification by WHO as critically important, being one of the last therapeutic options for certain diseases resulting from bacteria resistant to the drugs of choice (FAO/WHO, 2009). Thus, considering the presence of resistance to ciprofloxacin (5/7) and tetracycline (6/7) in these specific strains, colistin can be used as a treatment option for campylobacteriosis. However, the definition of intrinsic resistance for *Campylobacter* spp. means that most laboratories (CLSI and EUCAST) do not determine the cutoff points to classify the real susceptibility of this genus to colistin (Sorlózano-Puerto et al., 2018), which makes the results still speculative.

Due to the increasing resistance to fluoroquinolones in *Campylobacter*, macrolides, such as erythromycin and azithromycin, appear as drugs of choice in the treatment of human campylobacteriosis (Bolinger and Kathariou, 2017). Our results show that erythromycin was the second most efficient antibiotic in the evaluated strains, with a percentage of 34.3% (12/35) of resistance. Despite this, studies show an even greater efficiency with resistance rates ranging from 0 to 17.4% in several countries (Aksomaitiene et al., 2019; Melo et al., 2019; Otto et al., 2019; Tryjanowski et al., 2019; Elhadidy et al., 2020; Karama et al., 2020). In a contradictory way, it is possible to find high levels of resistance in isolated strains of chickens (Asuming-Bediako et al., 2019; Pillay et al., 2020). In Brazil, the reduced number of isolates resistant to erythromycin is probably due to decreasing exposure to antibiotics in poultry production after the implementation of a regulatory document that prohibits the use of macrolides in poultry farms.

Despite the low number of resistant strains to meropenem (3/35, 8.6%), compared to other classes, the result is alarming. Meropenem is among the drugs classified as critically important by WHO. Over the past 30 years, carbapenems have played a crucial role in clinical weaponry to treat serious infections in patients primarily affected by multidrug-resistant bacteria. However, the usefulness of this class of antibiotics is being compromised by the emergence of resistance in *Enterobacteriaceae* and especially in *Campylobacteriaceae*, being considered as a “nightmare” (Perez et al., 2016; Hagiya et al., 2018). In our study, two of these three strains have erythromycin as a treatment option, but one of them, in addition to resistance to meropenem, was also resistant to all other classes of tested antimicrobials. In cases of refractory infections by *Campylobacter*, in addition to combination therapy, the use of aminoglycosides is recommended as an alternative for severe cases (Trajkovska-Dokic et al., 2019).

There are statements by EFSA and CDC describing an impending global crisis that may result in a return to the pre-antibiotic era (CDC, 2015; EFSA, 2020). These serious concerns were catalyzed by the rapid increase in carbapenemase production among bacteria from the *Enterobacteriaceae* family (Bonomo et al., 2018). For severe infections caused by carbapenemase-producing Enterobacteriaceae, the possibility of treatment is the use of colistin, either singly or in combination with other antibiotics, which does not fit *Campylobacter* due to its intrinsic resistance (Halaby et al., 2013). Although resistance to carbapenemics in *Campylobacter* has not yet been well defined by the authorities, a study by Hagiya et al. (2018) suggested the existence of resistance due to subsequent exposure to the drug in a clinical situation. Our study demonstrates the first record of phenotypic resistance to meropenem in *C. jejuni* in Brazil.

Our study revealed that 71.4% (25/35) of the isolates were multidrug-resistant, and if we disregard colistin sulfate, this percentage drops to 37.1% (13/35). Proportions of less than 40% have been observed previously in *Campylobacter* spp. isolated from chickens (Liu et al., 2019; Melo et al., 2019). Values ​​similar to ours were identified by Di Giannatale et al. (2019) in Italy, with 66.15% of *C. jejuni* resistant to the same classes of antibiotics tested in our study (ciprofloxacin, tetracycline, and erythromycin).

### Antimicrobial Resistance Profile in the Biofilm Forms

The low efficacy of the drugs tested on the sessile structure had already been detected in other studies that report the influence of antimicrobials stimulating the acquisition of the sessile structure in several species of bacteria, which prevents the drug’s effect (Boehm et al., 2009; Kaplan, 2011; Teh et al., 2019).

Our findings make it clear that high concentrations of the drugs were necessary to control the viability of sessile *C. jejuni*. In addition, it was evident that each strain behaved differently regarding the increased concentration of the antimicrobial necessary to contain the sessile cells in biofilm. This strain-dependent characteristic was also identified by Teh et al. (2019) when analyzing the action of different classes of antimicrobials against seven strains of sessile *C. jejuni*. These authors also found that susceptible strains showed a higher production of biofilms compared to resistant strains. We determined that susceptible strains needed a higher concentration of antimicrobials to contain bacterial viability. This difference may be directly related to the nutrient substrate offered to the bacteria in the formation of the biofilm. In our study, we simulated the nutritional conditions of abiotic surfaces present in industry by adding CJ. This substrate stimulated the formation of a more stable, mature, and protein-rich biofilm than that produced only with the use of MH (Melo et al., 2017).

It is also possible that the penetration of antimicrobials by the matrix may have occurred more slowly, which favors the sessile cell, since the gradual exposure generates an adaptive phenotypic response that can potentially increase tolerance to the antimicrobial (Tseng et al., 2013). Another important factor concerns the high rate of mutation detected in sessile cells when compared to planktonic forms, which can contribute to the increase in antimicrobial resistance. Especially for *Campylobacter*, the genotypic variation resulting from recombination processes already represents an intrinsic factor of the bacteria in the free form and, interestingly, the sessile lifestyle besides promoting high mutation rates also contribute to the emergence of permanently hypermutable strains, as when biofilms are exposed ciprofloxacin, in which the presence of resistant mutants is significantly higher (Bae et al., 2014).

The discrepant results found for tetracycline in our study compared to other antibiotics may be related to the mechanism of action of this antimicrobial agent. This drug has a high diffusion capacity, which allows its use in the treatment of intracellular pathogens (Trabulsi et al., 1999), which probably can facilitate the penetration into the biofilm matrix. For example, tetracycline rapidly reached all cells uropathogenic *Escherichia coli* (UPEC) biofilms in a study conducted by Stone et al. (2002). In addition, this drug acts on the 30S portion of the ribosomes preventing protein synthesis (Trabulsi et al., 1999) and, according to Majtán et al. (2008), antibiotics acting on ribosomes can inhibit the formation of biofilms, interrupting the ability of bacteria to adhere. Considering that *C. jejuni* biofilms produced with CJ supplementation present a predominantly protein matrix (Melo et al., 2017) it is also possible that tetracycline has influenced the biofilm structure by increasing bacterial exposure.

### Effect of Antimicrobials on Biomass

Overall, all tested antimicrobials altered the biomass of *C. jejuni*, and in the presence of ciprofloxacin, colistin, erythromycin, and meropenem, this effect was positive, while for tetracycline, it was negative. The susceptibility to antimicrobials, present in the planktonic form of the F048 strain, especially to meropenem, induced a greater stimulation of biomass production, except for tetracycline.

Previous studies have reported that the presence of certain antibiotics influenced the formation of bacterial biofilm in a positive or negative way. For example, ciprofloxacin was shown to induce biofilm formation in *Escherichia coli* (Rafaque et al., 2020) and *Pseudomonas aeruginosa* (Soares et al., 2019). Tigecycline favored the formation of biofilm by *Staphylococcus epidermidis* (Weiser et al., 2016). In *Staphylococcus aureus*, vancomycin had a positive effect on the acquisition of biofilm form (Pasquaroli et al., 2013). On the other hand, the presence of sub-minimal inhibitory concentrations (MIC) of ciprofloxacin inhibited the biofilms of *Salmonella enterica* serovar Typhimurium (Majtán et al., 2008).

In our study, we detected significant fluctuations in the IFB of both strains treated on different surfaces and which did not follow a defined pattern. The exception was the treatment with meropenem and erythromycin, which intensified the F048 biomass (susceptible in the planktonic form) in all treatments and in the traditional methodology, respectively. Wild type strains, such as those used in our study, have specific gene expression modulation and modification systems that decisively alter the bacterial phenotype even in minimally different conditions (Teh et al., 2017). Our findings were similar to those by Teh et al. (2019) in *C. jejuni*, who observed that susceptible strains have a higher production of biofilms compared to resistant strains that reduce their production in the presence of the antibiotics ampicillin, nalidixic acid, erythromycin, rifampicin, and tetracycline. The significant increase in biomass in contact with meropenem has also been described by Navidifar et al. (2019) in susceptible *Acinetobacter baumannii* strains in planktonic form. The authors attribute this increase to the presence of persistent, metabolically dormant cells, usually present in biofilms. These cells, recently preceded in *C. jejuni*, are extremely tolerant to antibiotics without undergoing any genetic alteration (Morcrette et al., 2020). Another reason for this was suggested in a previous study, which proposed that some antibiotics can act as antagonists of the formation (and growth) of biofilm in low concentrations, agonists in higher concentrations and antagonists in even higher levels (Kaplan, 2011).

Contradictory to our study, Teh et al. (2019) found that, in a general analysis antimicrobials significantly reduce biomass of *C. jejuni*. In our study, this fact was exclusive for biofilms treated with tetracycline. In addition, only for the F639 strain there was no change in the IFB in biofilms produced in stainless steel and polyurethane, treated with meropenem and ciprofloxacin, respectively (**Figure 2**). The difference may be related to the use of sub-MIC concentrations used by the authors and the type of biofilm produced in our study (protein using CJ).

Kaplan (2011) suggested that there really are variations in the induction or not of biofilm production depending on the antimicrobial used, its concentration, the microorganism, and the composition of the polymeric matrix, performing agonist and antagonist functions and type of contact surface. All of these associated factors contributed to the variations detected in our study.

The surface composition can control the reactivity and binding of substrates, including bacterial extracellular polymers. Stainless steel is the most recommended material for food contact equipment and one of the factors that reinforce this indication is the low porosity and high resistance, in addition to its smooth surface (De Oliveira et al., 2019). However, some of the elements present in this material are favorable to bacterial adhesion, such as iron, manganese and calcium (Arnold and Silvers, 2000), which may justify the influence of stainless steel on the greater intensity of our biofilms treated with ciprofloxacin, colistin and erythromycin. It has been shown that *C. jejuni* is capable of forming biofilm on a variety of surfaces, including stainless steel, glass and plastics. Stainless steel promotes intensified production due to the high hydrophobicity of the bacterial cell surface that favors the initial cell fixation (Teh et al, 2016). Despite the polyurethane and polypropylene surfaces also showing hydrophobic properties (Iliadis et al., 2018; Vidács et al., 2018), in our work we observed a lower intensity in the biomass produced, with variation dependent on the strain and the antimicrobial. As the cell surface can be modulated by extrinsic characteristics and intrinsic factors, it is possible that exposure to the different conditions tested promoted a variation in hydrophobicity, altering the bacterial adhesion capacity, as identified by Moraes et al. (2019) in *Salmonella*. Especially for polyurethane, some studies have demonstrated its anti-biofilm effect on *Salmonella*, *S. aureus* and *P. aeruginosa* (Nazli et al., 2019; Brasão et al., 2021).

### Structural Change After Antimicrobial Treatment

The architecture of the biofilms produced showed the same characteristics for both strains in the respective treatments. The observed variations are consistent with the findings obtained in the MIC test and in the biomass assessment. The production of a more expanded and compact structure associated with the absence of bacteria exposed in biofilms treated with antimicrobials, shows that ciprofloxacin, colistin, erythromycin, and meropenem have an agonistic effect on the production of biofilms, causing a stimulus of greater matrix production in order to internally protect bacteria. As for tetracycline, the observed antagonistic effect was directly related to the destruction of the matrix and bacteria, making evident the greater susceptibility of the microorganism.

The variations in the architecture of the biofilms formed were also recorded by Turonova et al. (2015) in different strains of *C. jejuni* and by Melo et al. (2017) on different substrates, with multilayer structures, biofilms with a shape similar to “fingers”, with open and spongy ultra-structure, with and without the presence of pores. These reports make it clear that intrinsic and extrinsic factors can influence the structure of *C. jejuni* biofilm.

## Conclusion

Our results showed that the presence of susceptible strains to colistin may indicate a new treatment strategy, and that resistance to meropenem detected in three strains is alarming since it is the last therapeutic resource available currently. In biofilms, the high antimicrobial resistance matches the expanded and dense structure of the biomass, except for tetracycline. Susceptible strain in the planktonic form expressed a higher production of biofilms when in contact with the antimicrobial, which is consistent with the need for higher concentrations of the drug for its control compared to resistant strain, except for tetracycline. Stainless steel and polyurethane were the most and least suitable surfaces for the production of treated biomass, respectively. Interestingly, the exceptions pointed out for tetracycline in the biofilm form and for colistin in the planktonic form of *C. jejuni* affect possible control strategies and, conversely, the existence of resistant meropenem strains may represent an initial threat to public health.

## Data Availability Statement

The datasets generated for this study are available on request to the corresponding author.

## Author Contributions

DR wrote sections of the manuscript. CD and VS performed laboratory tests on Campylobacter in free form. AS and MV performed laboratory tests on Campylobacter in sessile form. JS performed laboratory tests on Campylobacter in sessile form in different materials. RP performed the phylogenetic analysis of the strains. GM and CM contributed to the statistical analysis of the data, translation of the manuscript and methodological guidance. JS wrote the final draft of the manuscript. RM revised the final draft of the manuscript, organized the database, performed the test in SEM, contributed to the conception and design of the study. All authors contributed to the article and approved the submitted version.

## Conflict of Interest

The authors declare that the research was conducted in the absence of any commercial or financial relationships that could be construed as a potential conflict of interest.
